# *Rab38* Mutation and the Lung Phenotype

**DOI:** 10.3390/ijms19082203

**Published:** 2018-07-27

**Authors:** Kazuhiro Osanai

**Affiliations:** 1 Department of Life Science, Medical Research Institute, Kanazawa Medical University, 1-1 Uchinada-Daigaku, Kahokugun, Ishikawa 920-0293, Japan; k-osanai@kanazawa-med.ac.jp; Tel.: +81-76-286-2211; Fax: +81-76-286-0980; 2 Department of Respiratory Medicine, Kanazawa Medical University, 1-1 Uchinada-Daigaku, Kahokugun, Ishikawa 920-0293, Japan

**Keywords:** *Rab38*, alveolar type II cells, pulmonary surfactant, lysosome-related organelle (LRO), biogenesis of lysosome-related organelle (BLOC), *chocolate* mouse, *Ruby* rat, Hermansky-Pudlak syndrome (HPS)

## Abstract

*Rab38* is highly expressed in alveolar type II cells, melanocytes, and platelets. These cells are specifically-differentiated cells and contain characteristic intracellular organelles called lysosome-related organelles, i.e., lamellar bodies in alveolar type II cells, melanosomes in melanocytes, and dense granules in platelets. There are *Rab38*-mutant rodents, i.e., *chocolate* mice and *Ruby* rats. While *chocolate* mice only show oculocutaneous albinism, *Ruby* rats show oculocutaneous albinism and prolonged bleeding time and, hence, are a rat model of Hermansky-Pudlak syndrome (HPS). Most patients with HPS suffer from fatal interstitial pneumonia by middle age. The lungs of both *chocolate* mice and *Ruby* rats show remarkably increased amounts of lung surfactant and conspicuously enlarged lysosome-related organelles, i.e., lamellar bodies, which are also characteristic of the lungs in human HPS. There are 16 mutant HPS-mouse strains, of which ten mutant genes have been identified to be causative in patients with HPS thus far. The gene products of eight of the ten genes constitute one of the three protein complexes, i.e., biogenesis of lysosome-related organelle complex-1, -2, -3 (BLOC-1, -2, -3). Patients with HPS of the mutant BLOC-3 genotype develop interstitial pneumonia. Recently, BLOC-3 has been elucidated to be a guanine nucleotide exchange factor for Rab38. Growing evidence suggests that *Rab38* is an additional candidate gene of human HPS that displays the lung phenotype.

## 1. Introduction

Small GTPase proteins are intracellular monomeric proteins characterized by GTP-binding and GTPase activities. The Ras protein was discovered first, and the discovery of other proteins soon followed. They form a *Ras* superfamily, which contains more than 150 members. The *Ras* superfamily is now considered to comprise three major gene families (*Ras*, *Rho*, and *Rab*) and other families. These proteins regulate essential cell functions, e.g., intracellular vesicle trafficking, exocytosis/endocytosis, cell growth/differentiation, and cytoskeletal configuration [1,2,3,4]. In organisms ranging from yeast to mammals, these proteins contain several consensus domains that contribute to the interaction with guanine nucleotides. Most of these proteins also contain unique lipid modifications at cysteine residue(s) in their carboxyl terminals, e.g., prenylation, and in *Ras*, palmitoylation as well. The *Rab* family, which contains over 60 members, is the largest family in the *Ras* small G protein superfamily. Their gene products (Rabs) localize to distinct cell organelles, and most of the proteins exist in either a membrane-associated form or a cytosolic form [2,5]. The Rab family proteins participate in intracellular vesicle trafficking between defined intracellular compartments [5]. Many Rab proteins, such as Rab1, Rab2, Rab4-14, Rab18, Rab20, Rab22, Rab24, Rab28, and Rab30, are ubiquitously expressed in numerous tissues and cells [2]. However, expression of some Rab proteins is highly specific depending on the cell type (endothelium, mesenchyme, or epithelium) and cell state (differentiated, activated, or polarized). Rab3, Rab15, Rab17, Rab19, Rab23, Rab25, Rab26, Rab27, Rab32, and Rab38 belong to these highly-specific Rab proteins [2]. Indeed, partial characterization of these Rab proteins was carried out from information about their expression in specific cells and intracellular localization [6,7].

**Figure 1:**
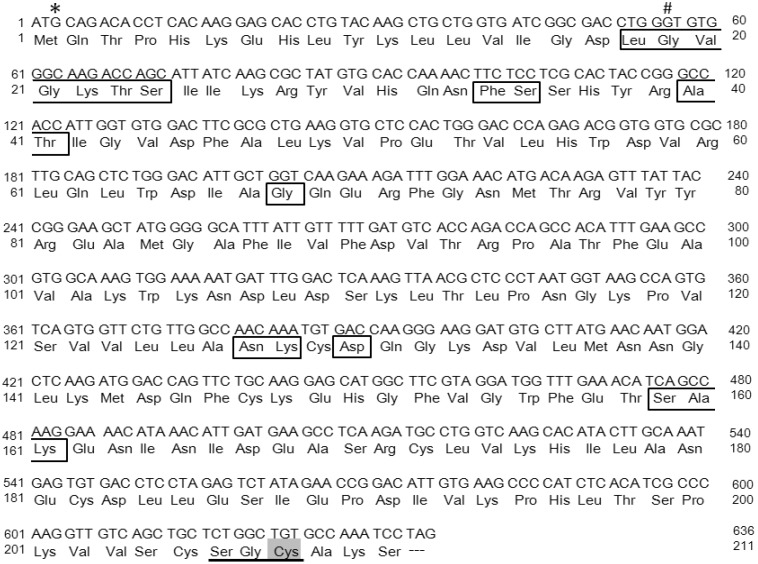
The cDNA sequence and the predicted amino acid sequence of mouse *Rab38*. *: In *Ruby* rat, G is converted to A, deleting the initiation codon to abolish translation. #: In *chocolate* mice, G is converted to T, and Gly changes to Val. The underlined amino acid sequence is a putative lipid modification site and Cys (half-toned) is modified with geranylgeranyl residue. Boxed amino acids are GTP/Mg^++^ binding sites [20].

Rab38 was discovered relatively late in the Rab small GTPase family and its expression is tissue-specific [8,9,10,11]. Originally, Rab38 cDNA was discovered from a rat lung cDNA library and was recognized as a novel *Rab* small GTPase protein [12]. It has been recorded in Genbank under accession no. M94043. Later, Rab38 was also isolated from a human melanoma cDNA library [13]. Analysis of the cDNA predicted a molecular weight of 24-kDa calculated from the deduced amino acid sequence (Figure 1). The analysis of the cDNA also indicated substantial similarity with other Rab proteins, especially Rab32 and Rab29. The NCBI Protein BLAST indicated that Rab32 shared 75% identity with Rab38 in terms of amino acid sequence, and Rab29 shared 52%. Expression of Rab38 mRNA is significantly different in various rat organs [14]. Expression is highest in lung, then skin, followed by the stomach, kidney, and liver. Rab38 is highly expressed in melanoma and causes specific antibody response in melanoma patients [15,16]. Rab38 has been implicated in frontotemporal dementia [17], and Parkinson’s disease (PD) via its interaction with leucine-rich repeat kinase 2 (LRRK2) [18,19]. Freshly isolated alveolar type II cells had higher mRNA expression than total lung, but the cells rapidly lost mRNA expression on culture in plastic culture dishes [14]. Alveolar type II cells that were isolated and cultured in plastic dishes overnight did not retain a significant signal of mRNA expression. There was no significant level of Rab38 mRNA expression in alveolar macrophages, A549 cells, COS-7 cells, and 293 cells [14]. This organ-specific expression was also confirmed by Western blot analysis. Thus, it is evident that Rab38 expression is tissue-specific and not ubiquitous.

## 2. Hermansky-Pudlak Syndrome

### 2.1. Hermansky-Pudlak Syndrome and the Lung Phenotype

HPS affects 1 in 500,000 to 1,000,000 individuals in the world [21,22]. However, in Puerto Rico, it affects 1 in 1800 people. Patients with HPS-1, -2, and -4 develop pulmonary fibrosis in their middle-age; it is progressive with age, and no curative treatment is available. This rare autosomal recessive disease is considered to be closely associated with intracellular trafficking relating to lysosome-related organelles (LROs) [21,23,24,25]. These LROs include melanosomes in melanocytes, dense granules in platelets, and lamellar bodies in alveolar type II cells. Patients with HPS clinically show oculocutaneous albinism and bleeding diathesis and based on the genetic background, other abnormalities, such as progressive pulmonary fibrosis, granulomatous colitis, and renal impairment [21,25]. Pulmonary fibrosis, or interstitial pneumonia, is the most critical complication and arises in patients with HPS in their middle-age, progressing to death without any effective therapy. Lungs of patients with HPS show a histopathologically usual interstitial pneumonia (UIP) pattern hallmarked by larger alveolar type II cells engorged with giant lamellar bodies [26]. Those cells contain remarkably increased surfactant phospholipids. These abnormalities in alveolar type II cells, lamellar bodies, and lung surfactant appear to be closely associated with the lung pathology in patients with HPS.

### 2.2. Animal Models of Hermansky-Pudlak Syndrome

**Figure 2:**
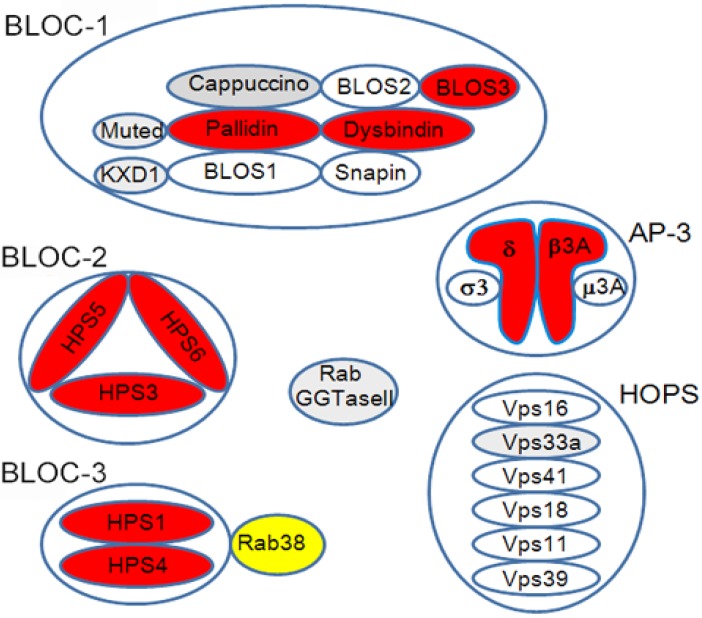
HPS protein complex. Red-colored molecules were identified in both mouse and human HPS, while gray-colored molecules were identified only in mice. Yellow-colored Rab38 is recognized as an HPS molecule in rats, but not completely in mice. BLOC3 functions as a guanine nucleotide exchange factor (GEF) for Rab38 [37].

There are at least sixteen mouse homologues of human HPS that show oculocutaneous albinism and bleeding diathesis [27] (Table 1). Among the sixteen mouse homologues, ten distinct mouse genotypes of HPS have been identified in humans (HPS1-10) thus far. In rats, only the Rab38-deficient rats carrying the *Ruby* phenotype, i.e., oculocutaneous albinism and bleeding diathesis, have been identified as a rat model of HPS, including Fawn-hooded rats, Tester-Moriyama rats, and Long Evans Cinnamon (LEC) rats [28,29,30]. The gene products identified in these animals and patients with HPS have been revealed to participate in intracellular vesicle trafficking relating to LROs, since they constitute several protein complexes, e.g., adaptor protein-3 complex, BLOC-1, -2, -3, or homotypic fusion and vacuole protein sorting (HOPS) [25,27,31] (Figure 2). HPS genotypes relating to BLOC-3 components and AP-3β3A, i.e., *HPS1*, *HPS2*, and *HPS4*, are susceptible to the lung disease [32]. Melanocytes, platelets, and alveolar type II cells are differentiated cells that develop LROs, including melanosomes in melanocytes, dense granules in platelets, and lamellar bodies in alveolar type II cells [33,34,35]. There are other examples of specifically differentiated cells that perform specific functions with characteristic LROs (Table 2).

The similarity between the lung changes in the lungs of *chocolate* mice and *Ruby* rats strongly suggests that these lung phenotypes belong to an animal model of HPS and that the *Rab38* mutation is the underlying cause. The hydrophobic lung surfactant overloading in the lung tissue and the abnormal morphology of alveolar type II cells in *Rab38*-mutated animal lungs are consistent with those reported in the mouse model of HPS with established genetic abnormality [31,36] and in patients with HPS [26]. It is highly possible that the lung phenotype in the *Rab38*-mutated animals mimics the animal model of human HPS lungs.

### 2.3. BLOC-3 Is a Guanine Nucleotide Exchange Factor for Rab32/38

Molecular characterization of Rab38 was extensively studied in melanocytes and related cell lines that traffic melanin synthesizing enzymes to melanosomes, i.e., LROs. Rab38 and its closely-related counterpart, Rab32, coordinate melanogenic enzyme transport in these cells [38]. In melanocytes, Rab32/Rab38 play a pivotal role in endosomal trafficking of melanogenic enzymes between early/recycling endosomes and biogenesis of melanosomes. Melanosome maturation is characterized by four morphologically distinct stages [39]. For melanin synthesis, several melanogenic enzymes, including tyrosinase, tyrosinase-related protein (TRP) 1 and TRP 2, need to be selectively packaged into distinct transport vesicles/intermediates at recycling/early endosomes [40]. Rab32/Rab38 interacts with the ubiquitous machinery, BLOC-2, AP-1, and AP-3, to compose specialized cargoes and promote their motility, tethering, and fusion with specific stages of the maturing melanosomes [41]. Rab32 and Rab38 have both redundant and unique roles in the trafficking of melanogenic enzymes and overall melanosome biogenesis [41].

Similar to many other Rab proteins, Rab32/Rab38 cycles between a GDP-bound inactive form and a GTP-bound active form, and require several regulatory proteins. VPS9-ankyrin-repeat protein (VARP) is an effector molecule of Rab32/Rab38 and is ubiquitously expressed [42]. The Rab32/Rab38-VARP complex regulates the function and recycling of VAMP7 (vesicle-associated membrane protein 7) on the melanosome membrane [43]. Interaction of myosin Vc and Rab32/Rab38 regulates trafficking of melanosomal cargoes carrying TRP1 and VAMP7 to the melanosome [44]. BLOC-2 has been shown to be an effector for Rab32/Rab38 to target recycling tubular carriers containing the melanogenic enzymes to the melanosome [45]. RUN (RPIP8/UNC-14/NESCA) and TBC (Tre-2/Bub2/Cdc16)-domain-containing protein 1 (RUTBC1) is a GTPase-activating protein (GAP) for Rab32/Rab38 [46]. The function of BLOC-3 has been elucidated to function as a Rab32/Rab38 guanine nucleotide exchange factor (GEF) [37]. Thus, BLOC-3 and RUTBC1 are important molecular switches of Rab32/Rab38 in melanocytes.

## 3. Expression of Rab38 in the Lung

### 3.1. Selective Expression of Rab38 in the Lower Respiratory Tract Epithelium

To elucidate the expression of the native Rab38 protein in the lung tissue, as well as its localizations to distinct lung cells and the intracellular organelles [12], an affinity-purified rabbit anti-rat Rab38 polyclonal antibody was generated using a synthetic peptide designed based on a C-terminal amino acid sequence [47]. In Triton X-114 phase separation of alveolar type II cells, most Rab38 was detected in the Triton X-114-extracted phase and only a trace amount was detected in the water-soluble phase [48]. This result strongly suggested that most Rab38 was present as a hydrophobic protein in the cells. Immunohistological examination of rat lung tissues with the anti-Rab38 antibody revealed specific immunostaining of many alveolar type II cells, and the terminal bronchial epithelial cells [12]. Other cells such as alveolar type I cells, alveolar macrophages, vessels, smooth muscles, and connective tissues, did not show significant immunoreactivity. Specific localization of *Rab38* mRNA in alveolar type II cells and terminal bronchial epithelial cells was clearly shown by in situ hybridization [12]. Thus, it became evident that Rab38 localized to the lower respiratory tract epithelium in the rat lung, especially at alveolar type II cells.

Clara cells are the dominant terminal bronchial epithelial cells in the rat lung, as more than 80% of terminal bronchial cells are Clara cells [49]. Therefore, most of the distal bronchial epithelium showing predominantly positive signals for Rab38 protein and mRNA must be Clara cells. Clara cells and alveolar type II cells have common functions in the terminal respiratory tract [50,51]. Both cells synthesize lung surfactant even though Clara cells do not have lamellar bodies, which are characteristic secretory granules storing lung surfactants. Although the two cells share common functions in lung surfactant metabolism, their morphological characteristics are quite different. Clara cells do not fully process pro-surfactant protein B (pro-SP-B) and do not secrete surfactant phosphatidylcholine [52]. Clara cells do not produce surfactant protein C (SP-C), an extraordinary hydrophobic apolipoprotein. SP-C expression is used as a specific marker for alveolar type II cells [53].

### 3.2. Intracellular Localization of Rab38 in Alveolar Type II Cells

Alveolar type II cells are often isolated from the excised rat lungs using porcine pancreatic elastase digestion and subsequent metrizamide density-gradient centrifugation [54,55,56]. A dounce glass homogenizer was used for homogenization of the isolated rat alveolar type II cells to preserve cell organelles. The homogenates were fractionated by either centrifugation with differential centrifugal forces or by sucrose density-gradient ultracentrifugation. In the centrifugal fractionation, positive immunoblot bands were observed in the total alveolar type II cell lysate, heavy vesicles, light vesicles, and cytosol fraction, whereas the nuclear fraction was negative [14]. The strongest signal was observed in the heavy vesicles. According to the previous studies that used disintegrated alveolar type II cells [54], it is likely that the heavy vesicles are dominantly rich in cell membranes, mitochondria, lysosomes, and multivesicular bodies, and the light vesicles are rich in microsomes, Golgi stacks, and lamellar bodies. In the sucrose density-gradient fractionation, the predominant positive signal was observed in a 0.9 molar (M) fraction in which the homogenized cell organelle sample was loaded. No specific signal was observed in the 0.4 M sucrose fraction that was compatible with the lung surfactant-rich fraction. These results indicated that Rab38 was not specifically enriched in lamellar body fractions, but associated with heavier organelles in alveolar type II cells. The lamellar bodies are distinct storage granules in alveolar type II cells containing lung surfactant components, such as phospholipids, neutral lipids, and surfactant apoproteins [57]. Subcellular localization of GST-tagged Rab38 was investigated by a confocal-laser scanning microscopy in COS cells and A549 cells after transfection [14]. GST-tagged Rab38 distributed throughout the cytoplasm with perinuclear concentration, showing a similar pattern to endoplasmic reticulum (ER) and Lamp-1 [14]. In isolated rat alveolar type II cells, native Rab38 partly colocalized with surfactant protein B [12]. However, it seemed unlikely that the Rab38 protein specifically localized to lamellar bodies.

It is of interest that after isolation alveolar type II cells rapidly lose their differentiated function, i.e., the synthesis of surfactant components [54,55]. Upon isolation, alveolar type II cells rapidly lose expression of Rab38 similarly to the case of lung surfactant [14]. Our previous study indicated that the lamellar body fraction derived from alveolar type II cells was not enriched with Rab38, and that Rab38 did not colocalize with surfactant protein A (SP-A) in double-immunostaining of rat alveolar type II cells in primary culture [12]. As described later in detail, lamellar bodies are characteristic organelles in alveolar type II cells and are storage granules for lung surfactant components. Upon appropriate stimulation, lamellar bodies undergo regulated secretion [55]. While Rab38 is specifically expressed in the alveolar type II cells that produce lung surfactant components, Rab38 was not abundant in the lamellar body fraction [12]. Thus, although Rab38 is possibly involved in some specialized function of alveolar type II cells, it is not clear whether Rab38 takes part in the biogenesis, transport, exocytosis, or endocytosis of lung surfactant.

## 4. Pulmonary Surfactant is Stored in Lamellar Bodies, i.e., Lysosome-Related Organelles, in Alveolar Type II Cells

### 4.1. Pulmonary Surfactant Is a Complex of Lipids and Apoproteins

Pulmonary surfactant consists of a complex of several lipids, mainly phosphatidylcholine, and four surfactant proteins (SP-A, -B, -C, and -D). Alveolar type II cells are the primary source for the synthesis of pulmonary surfactant components, which they transport and store in lamellar bodies, and secrete into the alveolar space to stabilize alveolar structure through the surface tension-lowering activity of lung surfactant [58]. However, SP-D is exceptional. After synthesis and transport to the Golgi apparatus, SP-D is not routed to the lamellar bodies but is constitutively secreted [59]. Thus, most SP-D exists in the alveolar lumen. Electron microscopic study demonstrated a picture of exocytosis of lamellar bodies and, hence, lamellar bodies are regarded as secretory granules. The composition of lung surfactant derived from a lamellar body fraction, which is purified by lung or cell homogenization and subsequent sucrose density-gradient ultracentrifugation, is similar to that collected from bronchoalveolar lavage (BAL) fluid. Hence, lamellar bodies are regarded as storage granules of lung surfactant [60]. Accordingly, it is likely that newly-synthesized surfactants are routed to the lamellar bodies to be stored, and then secreted upon the trigger of appropriate stimuli. While there are two major pathways for secretory proteins, i.e., the constitutive and the regulated secretion pathways, a single route for intracellular transport of lung surfactant components was postulated; all surfactant components except for SP-D are routed to and stored in the lamellar bodies [61]. However, there is some controversy about the classical simple route of intracellular trafficking of lung surfactant [55,62]. Sufficient evidence has accumulated to show that individual surfactant components take independent cellular trafficking pathways.

### 4.2. Blocking of the Intracellular Transport Pathway via the Golgi Apparatus

The intracellular transport routes of several lung surfactant components were investigated utilizing brefeldin A, which blocks the Golgi-mediated intracellular trafficking. Brefeldin A, a fungal metabolite derived from *Eupenicillium brefeldianum*, causes reversible disruption of the Golgi apparatus, but does not significantly affect endocytosis and lysosome function [63]. Brefeldin A blocks binding of the ADP-ribosylation factor (ARF) onto budding vesicles from the Golgi apparatus [64]. Brefeldin A stops protein transport from the endoplasmic reticulum to the Golgi apparatus. Thus, the antibiotics are a useful biological tool to study the function of the Golgi apparatus in vesicular trafficking. As expected, brefeldin A effectively disassembles the Golgi apparatus in alveolar type II cells [56]. Brefeldin A changed the GM130 (Golgi resident marker) localization pattern from a perinuclear pattern to a broad cytoplasmic pattern, but did not affect the intracellular localization pattern of BiP/GRP78 (ER resident marker). SP-B can be used as a resident marker protein for lamellar bodies [33]. SP-B is enriched in lamellar bodies that are LROs and the biogenesis of LROs is closely associated with endosome and lysosome functions [33]. As expected, brefeldin A did not affect the SP-B localization pattern exhibiting a large granular pattern in the cytoplasm [65].

### 4.3. Surfactant Phosphatidylcholine Transport Bypasses the Golgi-Dependent Pathway

Intracellular transport of newly-synthesized [^3^H]phosphatidylcholine to lamellar bodies was investigated using radiolabeling of isolated rat alveolar type II cells with [^3^H]choline chloride, subsequent cell homogenization and sucrose density-gradient ultracentrifugation [56]. Newly-synthesized [^3^H]phosphatidylcholine was retained in the cells but not secreted into the media without a secretory agonist. The [^3^H]phosphatidylcholine accumulated into the lamellar body fraction, i.e., 0.4 M sucrose fraction, in a time-dependent fashion, and brefeldin A did not affect the accumulation of [^3^H]phosphatidylcholine into the lamellar body fraction. Brefeldin A also did not affect the agonist-stimulated secretion of [^3^H]phosphatidylcholine from alveolar type II cells. In contrast, brefeldin A completely inhibited secretion of newly synthesized [^35^S]lysozyme from the cells, indicating that brefeldin A effectively inhibited protein transport. These results indicate that after being newly synthesized in the endoplasmic reticulum [^3^H]phosphatidylcholine transport to the lamellar bodies is not affected in presence of brefeldin A and, hence, bypasses the Golgi-dependent route in alveolar type II cells [56].

### 4.4. Surfactant Protein-A Undergoes Constitutive Secretion and Reuptake

In a study of SP-A knockout mice, it was revealed that SP-A is more important for innate immune function against several pathogens rather than for surface tension-lowering function [66,67]. SP-A is not necessary for the biogenesis of lamellar bodies but is required for the structural formation of tubular myelin formed from secreted lamellar bodies in the alveolar lumen. SP-A is not specifically enriched in the lamellar body but is mainly present in the alveolar lumen. However, SP-A regulates the secretion, configuration, and uptake/recycling of pulmonary surfactants [28,68,69]. Both SP-A and SP-D are lung collectins, i.e., collagenous carbohydrate binding proteins (C-type lectins), and show an affinity for various carbohydrates and lipid moieties expressed on several pathogens [70]. Both SP-A- and SP-D-deficient mice show normal appearance of lamellar body organelles in alveolar type II cells [69,71]. Intracellular transport of newly-synthesized SP-A was investigated using radiolabeling of isolated rat alveolar type II cells with [^35^S]methionine [55]. SP-A was purified from culture media, cell lysate, and lamellar body fraction using immunoprecipitation with a mouse anti-rat SP-A monoclonal antibody. A majority of newly synthesized [^35^S]SP-A was constitutively secreted, then some of this compound was endocytosed and incorporated into the lamellar bodies. The secretion of [^35^S]SP-A was completely inhibited in the presence of brefeldin A. These findings indicate a pathway of constitutive secretion and endocytosis of SP-A leading to accumulation into lamellar bodies in alveolar type II cells in primary culture [55].

### 4.5. Surfactant Protein-B Is Transported to Lamellar Body via the Golgi-Dependent Pathway

SP-B plays an essential role in alveolar surface tension-lowering activity, and is also associated with lamellar body biogenesis [72]. Patients with congenital SP-B deficiencies and SP-B knockout mice immediately develop severe respiratory insufficiency and die soon after birth [73,74]. SP-B is translated as a 40–42 kDa of preproprotein and is cleaved in several proteolytic steps to form a mature peptide consisting of 79 amino acids [75]. The mature SP-B is inserted into phospholipid head groups to stabilize phospholipid films and also enhance the rate of phospholipid spreading at the air-liquid interface [57,58]. Intracellular transport of newly-synthesized SP-B was investigated using radiolabeling of rabbit alveolar type II cells with [^35^S]methionine [65]. After cell homogenization, lamellar body fraction was isolated using the sucrose density-gradient ultracentrifugation. SP-B was collected by immunoprecipitation from cell lysate, the lamellar body fraction, and culture media using a mouse anti-pig SP-B monoclonal antibody, which showed good cross-reactivity with rabbit SP-B. Newly-synthesized [^35^S]SP-B was present in the cell lysate and the majority of the mature [^35^S]SP-B was found in the lamellar body fraction. Only a trace amount of mature [^35^S]-SP-B was found in the media. Brefeldin A, the Golgi apparatus inhibitor, arrested the proteolytic steps of [^35^S]pro-SP-B and its translocation to the lamellar body. The results provided straightforward evidence that newly synthesized SP-B was transported to and stored in the lamellar body via Golgi apparatus-dependent processing [65]. It is noteworthy that this SP-B transport pathway is independent of those of phosphatidylcholine or SP-A. In summary, each surfactant component takes independent pathways to the lamellar body rather than a single common pathway. These results suggest that lamellar bodies are not derived from the Golgi apparatus.

## 5. *Rab38*-Mutated Mice Show Abnormality in Homeostasis of Pulmonary Surfactant and Alveolar Architecture

### 5.1. Chocolate Is a Mouse Phenotype of a Rab38 Mutation

The *chocolate* mutation, which was discovered at the Jackson Laboratory in 1984, occurred in C57BL/6J background mice and shows autosomal recessive inheritance (MacPike A, Mobraaten LE. New coat color mutations, Dbr and cht. *Mouse News Lett* 1984; 70:86). Mice homozygous for *chocolate* (*cht*) have a rich dark brown coat compared to wild-type C57BL/6J black mice. The melanin level in hair follicles is decreased. The skin pigmentation shown in the ear and tail is also lighter. Although eye color difference is not easily identifiable in these mutants, histological evaluation showed a thinner pigment cell layer and choroid in the eyeball. Hence, these mice exhibit a phenotype of oculocutaneous albinism. A point mutation was identified in the *Rab38* gene in the mice [76]. Previous reports identified that Rab38 participated in the transport of melanogenic enzymes in melanocytes of the skin [39] and pigmented epithelial cells of the retina [77]. No color difference was visible between the heterozygous mice and wild-type mice, supporting the recessive inheritance of the phenotype. Neither breeding nor growth abnormalities were evident in the *chocolate* mice for the 24-week observation period. Genomic DNA sequencing showed a G146T transversion that must have changed glycine to valine (Gly19Val) in the conserved domain of GTP-binding activity near the N-terminus of the Rab38 protein (Figure 1). This mutation was initially expected to cause a disabled GTP-binding activity and, thus, to result in a dysfunction of the GTP/GDP molecular switch. However, the mutant gene product (Rab38*^cht^*) has not yet been characterized on a molecular level. Moreover, it is likely that the mutation in the *chocolate* locus might also cause lung disease, as well as oculocutaneous albinism, as high expression of the Rab38 mRNA and encoded protein was observed in both the skin and the lung tissues [14]. We characterized how the Rab38-G146T mutation affected the phenotype of the mouse lungs and what was the molecular basis for the mutant gene product (Rab38*^cht^* protein).

### 5.2. Aberrant Structure of Alveolar Tissues in Chocolate Mice

Histological examination of the lung tissue sections by light microscopy indicated that the *chocolate* mice developed enlarged alveolar spaces compared with the wild-type mice [48]. There was no significant inflammation. No significant difference was observed in the recovered bronchoalveolar lavage (BAL) fluids in terms of total protein amount and total cell number. In both groups, alveolar macrophages occupied more than 98% of the cells, and no remarkable difference was observed in the appearance of the cells. In the mutant mice, the lung volumes were significantly greater at both 12 and 24 weeks of age. The lung morphometry indicated a significantly smaller volume proportion of the alveolar wall (Vvw) and a significantly larger mean linear intercept (Lm), indicating an emphysematous change in alveolar airspace in the *chocolate* lung. The destructive index (DI) also showed a significant increase and increased with age. These morphological changes suggest that *chocolate* mice develop an alveolar structural abnormality similar to senile lungs [78]. Although *chocolate* mice showed increased DI, the remarkable destruction of alveolar structures, an abnormality characteristic of other emphysematous mice, such as cigarette smoke-inhaled mice, was not observed [79].

**Figure 3:**
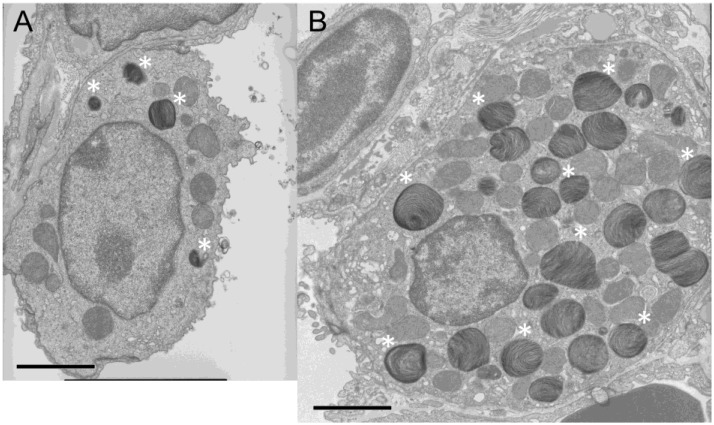
Electron microscopic image of an alveolar type II cell. (**A**) Wild-type C57BL/6J mouse. (**B**) *Chocolate* mouse. Some of the lamellar bodies are indicated by asterisks (*). The bar indicates 2.5 μm. Compared with wild-type mice, *chocolate* mice show enlarged alveolar type II cells filled with lamellar bodies of increased size and number.

Prestaining of the lung tissue sections prepared before electron microscopy showed numerous larger cytoplasmic bodies in alveolar type II cells in *chocolate* mice. By electron microscopic examination, these cytoplasmic bodies were identified as lamellar bodies of increased size and number, which filled the cytoplasm of the alveolar type II cells (Figure 3). However, despite the conspicuous changes in size and number of lamellar bodies, these lamellar bodies showed no apparent fine-structural abnormality.

### 5.3. Abnormal Lung Surfactant Homeostasis in the Chocolate Mice

Quantification of SP-A, SP-B, and SP-D in lamellar bodies, total lung homogenates, and BAL fluids was performed in both wild-type and *chocolate* mice [48]. No significant difference was observed in the amount of SP-A. In contrast, the amount of SP-B was increased in the total lung and lamellar body but was decreased in the BAL fluid in the *chocolate* mice. SP-D was detected only in the BAL fluid, but there was no significant difference in the amount of SP-D. Two-dimensional thin layer chromatography from *chocolate* mice lungs showed significantly increased phosphatidylcholine in the lamellar body and the total lung, but no significant difference in the BAL fluid. The *chocolate* mice also showed increased total phospholipid levels in the lamellar body and total lung, but no significant difference in the BAL fluid. Thus, in *chocolate* mice, hydrophobic components (SP-B and phosphatidylcholine) in lung surfactant were remarkably increased in the total lung and the lamellar body, but were not significantly different in the BAL fluid, while hydrophilic components (SP-A and SP-D) were not significantly changed.

### 5.4. Characterization of the Rab38^cht^ Protein

Triton X-114 phase partitioning was used to examine properties of the Rab38 protein expressed in the wild-type mouse. The Rab38 protein was found in both the aqueous and the detergent phases, although the protein in the detergent phase was more abundant [80]. However, when the same analysis of the Rab38*^cht^* mutant protein expressed in the *chocolate* mouse lung was performed, the Rab38*^cht^* mutant protein was exclusively found in the aqueous phase, and was only present in a cytosolic form, but not in a membrane-bound form [48]. This result was consistent with those of a previous paper in which a similar analysis was performed on melanocytes derived from *chocolate* mice [39]. These results suggested that Rab38*^cht^* is exclusively hydrophilic and appeared to be unable to bind to membrane components in cells.

Recombinant Rab38 protein is produced by employing the baculovirus and Sf9 cell protein expression system coupled with Ni^++^-charged affinity purification, and is used to characterize wild-type Rab38 protein (Rab38*^wt^*) and mutant Rab38 protein (Rab38*^cht^*) [14,48]. Unexpectedly, the recombinant mutant Rab38*^cht^* protein retained sufficient GTP-binding activity, although the mutation (G19V) existed in the conserved domain of the GTP-binding pocket near the N-terminus [81] (Figure 1). The recombinant Rab38*^cht^* protein radiolabeled with [^35^S]methionine is exclusively recovered in the aqueous phase, but not in the Triton X-114 detergent phase [14]. The Rab38*^cht^* protein was not radiolabeled with an isoprenoid precursor, [^3^H]mevalonate, indicating that the Rab38*^cht^* protein does not undergo posttranslational prenyl modification [48]. These results indicated that the mutant Rab38*^cht^* protein escapes posttranslational prenylation. The unprenylated Rab38 may fail to bind to the required membrane components and lose biological activity [82]. The precise mechanism of how such mutant proteins fail to undergo prenylation remains unclear. It may be possible that such mutants are unable to correctly interact with either the Rab escort protein (REP) or Rab geranylgeranyl transferase (RGGT), leading to escape from prenyl modification. Thus, we demonstrated that the Rab38*^cht^* mutant protein is inactive in membrane-binding activity due to failure in posttranslational prenyl modification, but not to GTP-binding inability.

### 5.5. Chocolate Mutation and the Oculocutaneous Lung Disease

Oculocutaneous albinism coupled with lung disease is closely associated with human HPS, a genetic disease that is clinically diagnosed when oculocutaneous albinism is complicated with bleeding diathesis, and not inevitably, but frequently, with pulmonary fibrosis. However, the clinical picture of HPS is heterogeneous depending on the heterogeneity of the causative genes [59]. In patients with HPS, the most serious problem is pulmonary fibrosis, i.e., interstitial pneumonia, which may cause death in middle-age [21,23,83]. The pulmonary pathology observed in the lungs of patients with HPS is consistent with that observed in the mouse models of human HPS, e.g., *pale ear* (*ep*), *pearl* (*pe*) double mutant mice. The *ep*/*ep*, *pe*/*pe* double mutant mice spontaneously, but slowly, developed interstitial pneumonia [84,85]. The underlying mechanism appears to be stress and apoptosis of alveolar type II cells resulting from excessive accumulation of lung surfactant [85]. The *ep*/*ep*, *pe*/*pe* double mutant mice developed integrated pulmonary abnormalities with conspicuous changes in alveolar type II cells. Interestingly, the alveolar type II cells and lamellar bodies were remarkably enlarged in the *ep*/*ep*, *pe*/*pe* double mutant mice, and the lung contained large amount of lung surfactant. Moreover, the airspaces of the *ep*/*ep*, *pe*/*pe* double mutant lungs contained age-dependent elevated numbers of inflammatory cells and foamy macrophages. All these features are similar to the lung pathology that is characteristic of patients with HPS [26]. However, each of the single mutant mouse shows subtle lung changes, such as foamy alveolar macrophages and enlarged alveolar type II cells with ceroid lipofuscins, and causes mild emphysematous pulmonary change, but not apparent pulmonary fibrosis [86]. Surprisingly, these single mutant mice are more prone to fibrotic lung changes induced by intratracheal instillation of low-dose bleomycin than control mice (C57BL/6J) [86].

The *chocolate* mice lack one of the major phenotypes of HPS, i.e., bleeding diathesis resulting from the platelet storage granule deficiency. No difference in the bleeding time was observed between wild-type and *chocolate* mice, as reported in an earlier paper [76]. Based on transmission electron microscopy, we found that platelets in the *chocolate* mice contained dense granules and that there was no morphological difference in platelets between wild-type and *chocolate* mice. A possible explanation may be that because the Rab38*^cht^* mutant is a full-length protein, the mutant protein may still be able to influence platelet storage granules to function. Another possibility is that there is some functional redundancy between Rab38 and other factors. For example, another member of the Rab family, Rab32, is closely related to Rab38 and may compensate for the deteriorated intracellular trafficking of melanogenic substances in *chocolate* melanocytes [39].

## 6. Rab38-Deficient Rats (*Ruby*) Are an Animal Model of Human Hermansky-Pudlak Syndrome

### 6.1. The Ruby Mutation Abolishes Translation of the Rab38 Gene in Rats

In contrast to many mouse models of HPS, only two animal models of HPS were reported in rats, i.e., Fawn-Hooded (FH) rats and Tester-Moriyama rats [29]. FH rats carry several other diseases, including systemic hypertension, pulmonary hypertension, renal failure, depression, and alcoholism, as well as oculocutaneous albinism and bleeding diathesis [30]. Pleiotropic effects of a single gene, *Ruby* (*R*), have been linked to oculocutaneous albinism and bleeding diathesis. *Ruby* is located on rat chromosome 1 near other FH loci, *Rf-1*, *Rf-2*, and *Bpfh-1*, which cause renal diseases and systemic hypertension. *Rab38* was shown to be consistent with the *Ruby* locus, leading to the idea that the *Ruby* rat and *chocolate* mouse genes are *Rab38* [29]. The point mutation in the initiation codon of *Rab38*, ATG to ATA, found in both FH and TM rats must completely abolish protein translation from the mutant allele (Figure 1). Using our anti-rat Rab38 antibody, we showed that *Ruby* rat lung completely lacked Rab38 protein expression [28]. Hence, it is likely that, in addition to oculocutaneous albinism, a platelet pool storage deficiency leading to a bleeding diathesis is caused by a complete deficit of Rab38. Thus, *Rab38* has been added as another candidate gene that will cause HPS in humans [23]. Since alveolar type II cells highly express Rab38, similar to melanocytes and platelets [14,21,24,25], it seems rational to hypothesize that Rab38 deficiency may cause the HPS lung phenotype that is closely associated with abnormalities in alveolar type II cell morphology and lung surfactant metabolism. Abnormal lung surfactant pooling and aberrant alveolar structures were observed in *chocolate* mice that carry another *Rab38* mutation [48]. *Chocolate* mice show oculocutaneous albinism, but lack bleeding diathesis. Hence, these mice are not considered as a complete animal model of HPS. Thus, it is not clear whether the *chocolate* lung phenotype is closely associated with lungs in HPS until similar lung abnormalities are clarified in *Ruby* rats.

### 6.2. Altered Homeostasis of Lung Surfactant in Ruby Rats

Long Evans Cinnamon (LEC) rats are also recognized as *Ruby* rats carrying the same *Rab38* mutation [28]. Toluidine blue staining of the LEC rat lung showed that LEC alveolar type II cells contained conspicuously larger cytoplasmic granules. A transmission electron microscopy revealed that LEC alveolar type II cells contained peculiar giant lamellar bodies [28]. These changes were similar to those observed in *chocolate* mice, but the extent of the changes was remarkably amplified. The amount of lung surfactant in total lung homogenate, the lamellar body fraction, and BAL fluid were compared between the control rats and the LEC rats. The LEC rats showed increased amounts of phosphatidylcholine in the lamellar body fraction and the lung homogenate, but no significant difference in phosphatidylcholine in the BAL fluid. The amounts of SP-A, SP-B, and SP-D were quantified by Western blotting. In the LEC rats, decreased levels of SP-A were observed in the lamellar body fraction, the lung homogenate, and the BAL fluid. Increased levels of SP-B were shown in the lamellar body fraction and the lung homogenate, but decreased levels of SP-B were shown in the BAL fluid. SP-D levels were not significantly different in the BAL fluids between the control and LEC rats. Real-time PCR using TaqMan gene expression assay was used to determine mRNA expression levels of SP-A, -B, -C, -D, and GAPDH. While surfactant protein expression levels were different between the control and the LEC rats, mRNA expression levels of SP-A, -B, -C, and -D in the lung tissues were not significantly different.

There are multiple intracellular signaling pathways for surfactant phosphatidylcholine secretion from alveolar type II cells, such as protein kinase A, protein kinase C, Ca^2+^/calmodulin-dependent protein kinase and so on [87]. [^3^H]choline chloride was added to freshly-isolated rat alveolar type II cells. No significant difference in [^3^H]phosphatidylcholine synthesis was observed between the control and the LEC rats. However, LEC alveolar type II cells indicated significantly lower basal secretion of [^3^H]phosphatidylcholine. In contrast, compared with the control alveolar type II cells, the agonist-induced secretion of [^3^H]phosphatidylcholine was surprisingly amplified in the LEC cells [28]. In the LEC alveolar type II cells, the order of the intensity of secretory stimulations was similar to that in the control cells, i.e., TPA > ATP > terbutaline. This result indicated that most signaling pathways are amplified in LEC alveolar type II cells. Thus, alveolar type II cells in LEC rats showed an aberrant secretion pattern of newly-synthesized [^3^H]phosphatidylcholine; suppressed basal secretion and enhanced agonist-induced secretion. As previously reported, the addition of human SP-A effectively inhibited TPA-induced secretions from both LEC and control alveolar type II cells. In contrast, there was no significant difference in liposome uptake between the control and LEC alveolar type II cells. Liposomes containing [^3^H]phosphatidylcholine were added to the cultured cells and incorporated [^3^H]phosphatidylcholine was analyzed [28]. Thus, Rab38 deficiency did not appear to affect liposome uptake by alveolar type II cells.

### 6.3. Morphological Changes of Alveolar Type II Cells in Ruby Rats Share Similarity with those in Human HPS Lungs

The histopathologic feature of the lung disease found in patients with HPS is a UIP pattern [26], in which alveolar septa showed conspicuous proliferation of alveolar type II cells undergoing characteristic foamy swelling/degeneration. Histochemical analysis showed that these peculiar alveolar type II cells were recognized as containing excessive phospholipid accumulation. Electron-microscopic examination showed the presence of numerous giant lamellar bodies, again suggesting cellular degeneration (i.e., giant lamellar body degeneration), caused by an excessive accumulation of lung surfactant. From these results, the presence of disordered lung surfactant metabolism in HPS alveolar type II cells was suggested. This phospholipid overloading and the peculiar morphological changes in alveolar type II cells observed in patients with HPS are similar to those found in the LEC rats [28] and in the *ep*/*ep*, *pe*/*pe* double mutant mouse models of HPS [84]. Thus, Rab38-deficient rats are an animal model of human HPS and develop aberrant lung surfactant homeostasis with peculiar lamellar body morphology. These results suggest that Rab38 deficiency will cause the HPS-type lung phenotype in addition to oculocutaneous albinism and bleeding diathesis.

### 6.4. Exogenous Gene Transfer of Rab38 Transgene into Ruby Rat Lungs

We constructed a replication-deficient recombinant adenovirus expressing Rab38 (Ad-Rab38) or as control lacZ (Ad-lacZ). Single endobronchial administration of Ad-lacZ into rat lungs effectively distributed into whole lungs and reached the terminal airway epithelium, which was evaluated by ex vivo staining of the excised lungs with lacZ stain [88]. Then, we investigated the effect of endobronchial administration of Ad-Rab38 on lung surfactant homeostasis in the Rab38-deficient LEC rat lungs [88]. The sizes of both enlarged alveolar type II cells and giant lamellar bodies were decreased close to those of normal lamellar bodies two weeks after Ad-Rab38 delivery. This result was similar to the previous report that the enlarged lamellar body phenotype was rescued by transiently-expressed EGFP-Rab38 in cultured FH rat alveolar type II cells in vitro [89]. Consistent with this result, lung surfactant phosphatidylcholine pooling was improved after the Ad-Rab38 delivery. The amount of phosphatidylcholine was significantly lower in the lung tissue and the lamellar body fraction in Ad-Rab38-treated LEC rats, but was not different in BAL fluids. The amount of SP-B detected by Western blotting was decreased in lamellar body fractions in Ad-Rab38-treated LEC rats, whereas the amount of SP-A was not significantly different. Thus, adenovector-mediated gene transfer of *Rab38* successfully ameliorated abnormal lung surfactant homeostasis in the lungs of rat HPS models in vivo. Our result suggests that endobronchial transgene delivery deserves further study as a possible therapeutic option for HPS lung disease. However, an adenovector provokes lung inflammation, which prevents a safe clinical application; an alternative delivery method, such as adeno-associated virus (AAV) vector, needs to be considered [90].

## 7. Conclusions

Similar to melanocytes and platelets, alveolar type II cells highly express Rab38. Spontaneous mutation of *Rab38* occurring in mice and rats cause abnormal lung surfactant homeostasis and development of peculiar giant lamellar bodies, i.e., lysosome-related organelles. This lung phenotype is shared between the rodent models of HPS and human HPS. Growing evidence suggests that *Rab38* is an additional candidate gene for the lung disease in human HPS.

## Figures and Tables

**Table 1:** Murine models of Hermansky-Pudlak syndrome and identified human homologs.

Mouse HPS	Mouse HPS Gene	Protein (Complex)	Human HPS Gene	Lung Disease in Human HPS (Ref)
Pale ear (*ep*)	*HPS1*	BLOC-3	*HPS1*	+ [22]
Pearl (*pe*)	*AP3B1*	AP-3	*HPS2*	+ [34]
Cocoa (*coa*)	*HPS3*	BLOC-2	*HPS3*	−
Light ear (le)	*HPS4*	BLOC-3	*HPS4*	+ [35]
*Ruby* eye-2 (*ru2*)	*HPS5*	BLOC-2	*HPS5*	−
*Ruby* eye (*ru*)	*HPS6*	BLOC-2	*HPS6*	−
Sandy (*sdy*)	*DTNBP1*	BLOC-1	*HPS7*	−
Reduced pigmentation (*rp*)	*BLOC1S3*	BLOC-1	*HPS8*	−
Pallid (*pa*)	*BLOC1S6*	BLOC-1	*HPS9*	+−
Mocha (*mh*)	*AP3D1*	AP-3	*HPS10*	−
Muted (*mu*)	*MUTED*	BLOC-1	−	
Cappuccino (*cno*)	*CNO*	BLOC-1	−	
Gunmetal (*gm*)	*RABGGTA*	Rab GGTase	−	
Buff (*bf*)	*Vps33A*	Class C VPS	−	
KXD1-KO	*KXD1*	BLOC1	−	
Ashen	*Rab27A*	Rab27A	−	
Chocolate (*cht*), *Ruby* (*R*) *	*Rab38*	Rab38	−	

*: *Ruby* (*R*) is a rat model of Hermansky-Pudlak syndrome. When lung disease is frequently present in human HPS, it is noted as ‘+’; ‘–‘ means not present: ‘+−‘ means sometimes present.

**Table 2:** Representative lysosome-related organelles (LROs).

LRO	Cells	Function	Affected Phenotype
Lamellar body	Alveolar type II cell	Storage of pulmonary surfactant	Interstitial pneumonia, Respiratory failure
Melanosome	Melanocyte	Melanin synthesis and transport	Albinism
Dense granule	Platelet, Megakaryocyte	Stopping bleeding	Bleeding diathesis
Lytic granule	Cytotoxic lymphocyte, Natural killer cell	Cell-mediated cytotoxicity	Immunodeficiency, Infectious susceptibility
Azurophil granule	Neutrophil, Eosinophil	Storage of proteases	Immunodeficiency, Infectious susceptibility
MHC class II compartment	B lymphocyte, Macrophage, Dendritic cell, Antigen-presenting cell	Intercellular recognition and communication	Immunodeficiency
Weibel-Palade body	Endothelial cell	Storage and secretion of hemostatic and proinflammatory substances	Bleeding diathesis

## Abbreviations

HPS	Hermansky-Pudlak syndrome
LRO	Lysosome-related organelle
BLOC	Biogenesis of lysosome-related organelle complex
SP	Surfactant protein
LEC	Long Evans Cinnamon
BAL	Bronchoalveolar Lavage
